# Prediction of Clinical Outcome in Endometrial Carcinoma Based on a 3-lncRNA Signature

**DOI:** 10.3389/fcell.2021.814456

**Published:** 2022-02-01

**Authors:** Hongmei Ding, Fei Jiang, Lifeng Deng, Juan Wang, Ping Wang, Mintao Ji, Jie Li, Weiqiang Shi, Yufang Pei, Jiafu Li, Yue Zhang, Zengli Zhang, Youguo Chen, Bingyan Li

**Affiliations:** ^1^ Deparment of Nutrition and Food Hygiene, Medical College of Soochow University, Suzhou, China; ^2^ Department of Obstetrics and Gynecology, The First Affiliated Hospital of Soochow University, Suzhou, China; ^3^ Department of Occupational and Environmental Health, School of Public Health, Medical College of Soochow University, Suzhou, China; ^4^ Department of Obstetrics and Gynecology, The Second Affiliated Hospital of Soochow University, Suzhou, China; ^5^ State Key Laboratory of Radiation Medicine and Protection, School of Radiation Medicine and Protection, Soochow University, Suzhou, China; ^6^ Department of Pathology, The First Affiliated Hospital of Soochow University, Suzhou, China; ^7^ Department of Epidemiology and Biostatistics, School of Public Health, Soochow University, Suzhou, China

**Keywords:** endometrial carcinoma, lncRNA signature, prognosis, diagnostic, biomarker

## Abstract

Endometrial carcinoma (EC) is one of the common gynecological cancers with increasing incidence and revived mortality recently. Given the heterogeneity of tumors and the complexity of lncRNAs, a panel of lncRNA biomarkers might be more precise and stable for prognosis. In the present study, we developed a new lncRNA model to predict the prognosis of patients with EC. EC-associated differentially expressed long noncoding RNAs (lncRNAs) were identified from The Cancer Genome Atlas (TCGA). Univariate COX regression and least absolute shrinkage and selection operator (LASSO) model were selected to find the 8-independent prognostic lncRNAs of EC patient. Furthermore, the risk score of the 3-lncRNA signature for overall survival (OS) was identified as CTD-2377D24.6 expression × 0.206 + RP4-616B8.5 × 0.341 + RP11-389G6.3 × 0.343 by multivariate Cox regression analysis. According to the median cutoff value of this prognostic signature, the EC samples were divided into two groups, high-risk set (3-lncRNAs at high levels) and low-risk set (3-lncRNAs at low levels), and the Kaplan–Meier survival curves demonstrated that the low-risk set had a higher survival rate than the high-risk set. In addition, the 3-lncRNA signature was closely linked with histological subtype (*p* = 0.0001), advanced clinical stage (*p* = 0.011), and clinical grade (*p* < 0.0001) in EC patients. Our clinical samples also confirmed that RP4-616B8.5, RP11-389G6.3, and CTD-2377D24.6 levels were increased in tumor tissues by qRT-PCR and *in situ* hybridization. Intriguingly, the *p*-value of combined 3-lncRNAs was lower than that of each lncRNA, indicating that the 3-lncRNA signature also showed higher performance in EC tissue than paracancerous. Functional analysis revealed that cortactin might be involved in the mechanism of 3-lncRNA signatures. These findings provide the first hint that a panel of lncRNAs may play a critical role in the initiation and metastasis of EC, indicating a new signature for early diagnosis and therapeutic strategy of uterine corpus endometrial carcinoma.

## Introduction

Globally, the incidence for uterine corpus endometrial carcinoma (UCEC) persistently increased with 1.3% per year from 2007–2016, in part due to continued declines in the fertility rate as well as increased obesity (Siegel et al., 2020). In China, the incidence of EC was also increasing from 2014, which ranked second in female reproductive malignancies on account of the increased risk factors such as diabetes and obesity (Chen et al., 2019). Although EC has a good prognosis with 5-year overall survival (OS) of 74–91%, the advanced or metastatic EC patients still have a poor prognosis due to tumor metastasis and poor differentiation (Piulats et al., 2017). Histological classification and the International Federation of Gynecology and Obstetrics (FIGO) staging system are the traditional treatment guideline and prognostic indicators (Pecorelli, 2009; Morice et al., 2016). However, distinct molecular characteristics have been demonstrated in the same stage and histology of cancers (Murali et al., 2014; Yang et al., 2016). With the development of precision medicine, a new therapeutic approach according to molecular profiling has been provided. In 2021, to improve outcomes of EC patients, molecular classification was recommended to select appropriate treatment regimens by the National Comprehensive Cancer Network (NCCN). Four molecular subgroups have been classified in 2013 based on the integrated genomic data of 373 endometrial carcinomas (Levine et al., 2013). Nevertheless, the integrated classification had limited application due to high expense and complex procedures. Therefore, identifying an efficient prognostic and diagnostic signature to guide clinical practice for EC is urgent.

Noncoding RNA was initially recognized as simply leaky transcription noise because they are not translated into proteins. However, numerous noncoding RNAs showed specific functions in cellular processes, as well as the dysregulation in human pathologies. Long noncoding RNA (lncRNA) is a class of noncoding transcripts with more than 200 nucleotides in length. Compelling studies reported that lncRNAs were associated with various human diseases including cancer by participating in biological processes widely (Schmitt and Chang, 2016; Peng et al., 2017; Yang et al., 2019). Meanwhile, accumulating evidence supported the potential ability of lncRNAs as cancer biomarkers (Lim et al., 2019; Li et al., 2020; Xie et al., 2020) and the prognostic value of lncRNAs (He et al., 2014; Sun et al., 2021). For example, Liu et al. systematically discussed the EC-related lncRNAs and their roles in different cancer hallmarks, including tumor growth, metastasis, maintenance of cancer stem cells, and chemoresistance (Liu H et al., 2019). Until now, some biomarkers for EC have been identified using gene expression profile data. However, these models are limited to a specific stage or grade of EC. For example, one study identified a prognostic model for patients with early-stage EC using reverse-phase protein arrays (Yang et al., 2016). Others found a prognostic value of immune, metabolic, or autophagy-related coding and noncoding lncRNAs for EC (Ouyang et al., 2019; Gao et al., 2020; Li and Wan, 2020; Wang et al., 2021). However, given the heterogeneity of EC and the complexity of lncRNAs, a panel of lncRNA biomarkers might be more precise and stable for predicting prognosis rather other one lncRNA. Therefore, it is timely to investigate the new lncRNA biomarkers by combining The Cancer Genome Atlas (TCGA) data with UCEC-specific data.

In the present study, we obtained the lncRNA expression profile and clinical information of UCEC patients from the datasets of TCGA project. By bioinformatic approaches, a potential 3-lncRNA signature was identified in EC, and the association between the signature and clinical characteristics was confirmed. Furthermore, clinical samples were used to demonstrate that 3-lncRNA signature has a much better performance than independent 3 lncRNAs, providing a new signature for early diagnosis and therapeutic strategy of EC.

## Results

### Identification of Differentially Expressed Long Noncoding RNAs Associated with Uterine Corpus Endometrial Carcinoma from The Cancer Genome Atlas

**FIGURE 1:**
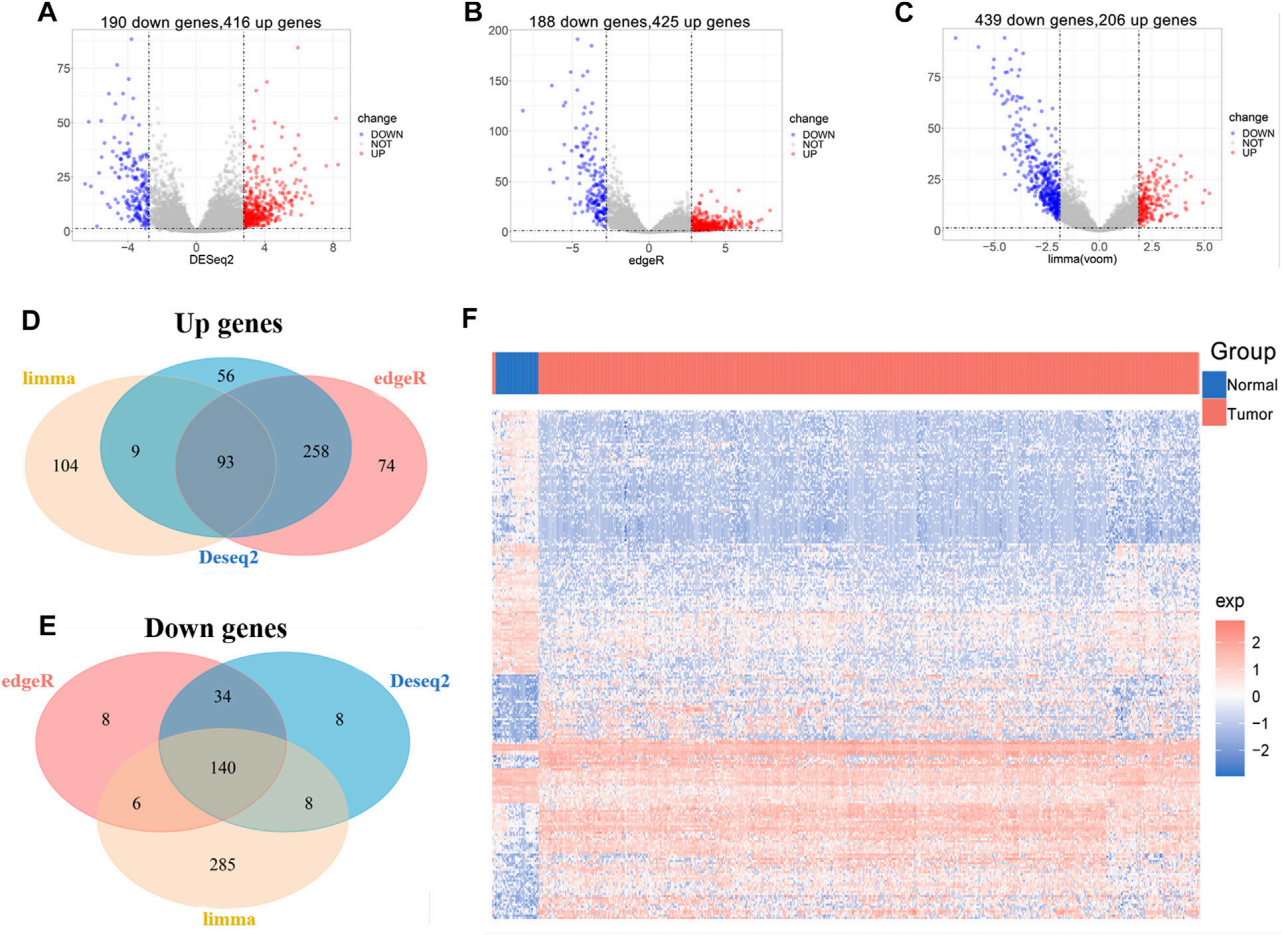
Identification of differentially expressed lncRNAs associated with UCEC from TCGA. **(A-C)** Volcano plot of DElncRs (different expressions of lncRNAs) between tumors and normal tissue by using “DEseq” (A), “edgeR” (B), and “limma” (C) R package. An absolute log2 fold change (FC) > mean (abs (logFC)) + 2*sd (abs (logFC) and an adjusted *p*-value of < 0.05 cutoff was used to defined DElncRs. **(D)** Venn diagram of upregulated lncRNAs. **(E)** Venn diagram of downregulated lncRNAs. **(F)** The heat map of DElncRs in TCGA–UCEC.

We obtained lncRNA expression profiles in 548 UCEC tissues and 35 normal tissues from TCGA datasets to screen DElncRs. To obtain reliable and stable results, lncRNA expression data were downloaded and performed using “DEseq,” “edgeR,” and “limma” R package separately in the R software (Figures 1A–C), and the intersections were acquired. Among the acquired lncRNAs, a set of 233 lncRNAs, including 93 upregulated and 140 downregulated, was abundantly expressed in all the uterine corpus endometrial carcinoma (Figures 1D–F, Supplementary Table S1). These results indicated the role of differentially expressed lncRNAs in the initiation and progression of uterine corpus endometrial carcinoma.

### Validation of Prognostic Long Noncoding RNA Signature

**FIGURE 2:**
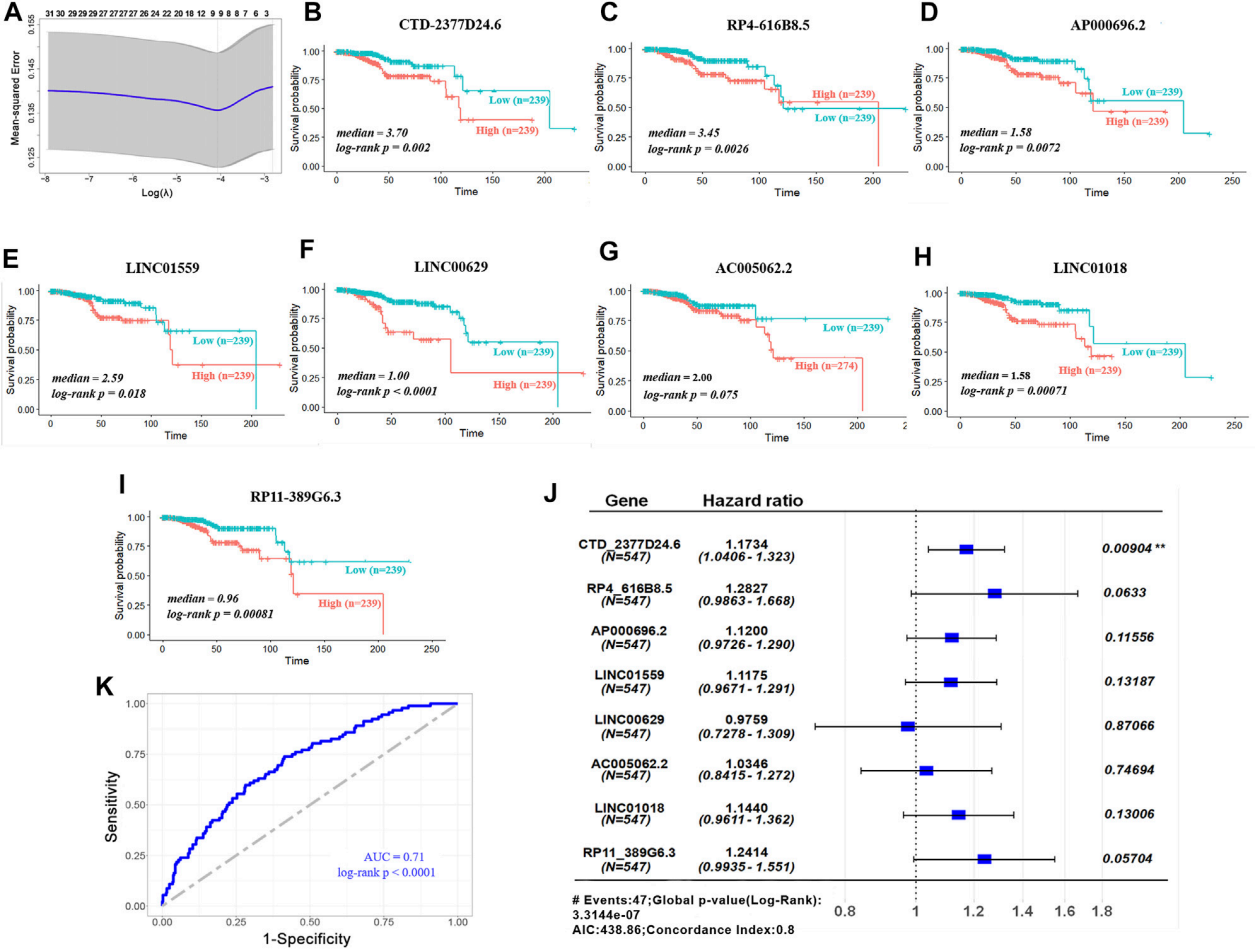
Validation of prognostic lncRNA signature. **(A)** LASSO coefficient profiles of 31 lncRNAs. **(B-I)** Overall survivals of the 8 lncRNAs in TCGA–UCEC. Kaplan–Meier curves were plotted for overall survival in patients with either high or low levels of lncRNAs, classified according to median of each lncRNA. *p* values were calculated using log-rank test. Y-axis, overall survival probability; X-axis, time in months. **(J)** Hazard ratios of the signature for overall survival of 8 lncRNAs. **(K)** ROC curve analysis of 8-lncRNA model for survival prediction of UCEC.

Subsequently, univariate Cox regression analysis was conducted to estimate the prognostic relationship between DElncRs and EC patient OS, and 31 prognostic lncRNAs were obtained with a *p* < 0.05 (Figure 2A). Furthermore, to minimize prediction errors, 9 lncRNAs were screened out using the LASSO regression method. Kaplan–Meier survival curves were used to further analyze the relationship between the 9 lncRNAs and the OS of EC patients. Ultimately, 8 lncRNAs were identified to be related with OS (Figures 2B–I). Multivariable Cox regression analysis revealed the hazard ratios of 8 lncRNAs for OS of endometrium carcinoma (Figure 2J). The area under the ROC curve (AUC) for OS was 0.71 (Figure 2K). These results implied that the 8-lncRNA model could efficiently identify the risk of EC prognosis.

### Assessment of Prognostic Risk in Uterine Corpus Endometrial Carcinoma Patients Using a 3-Long Noncoding RNA Model

**FIGURE 3:**
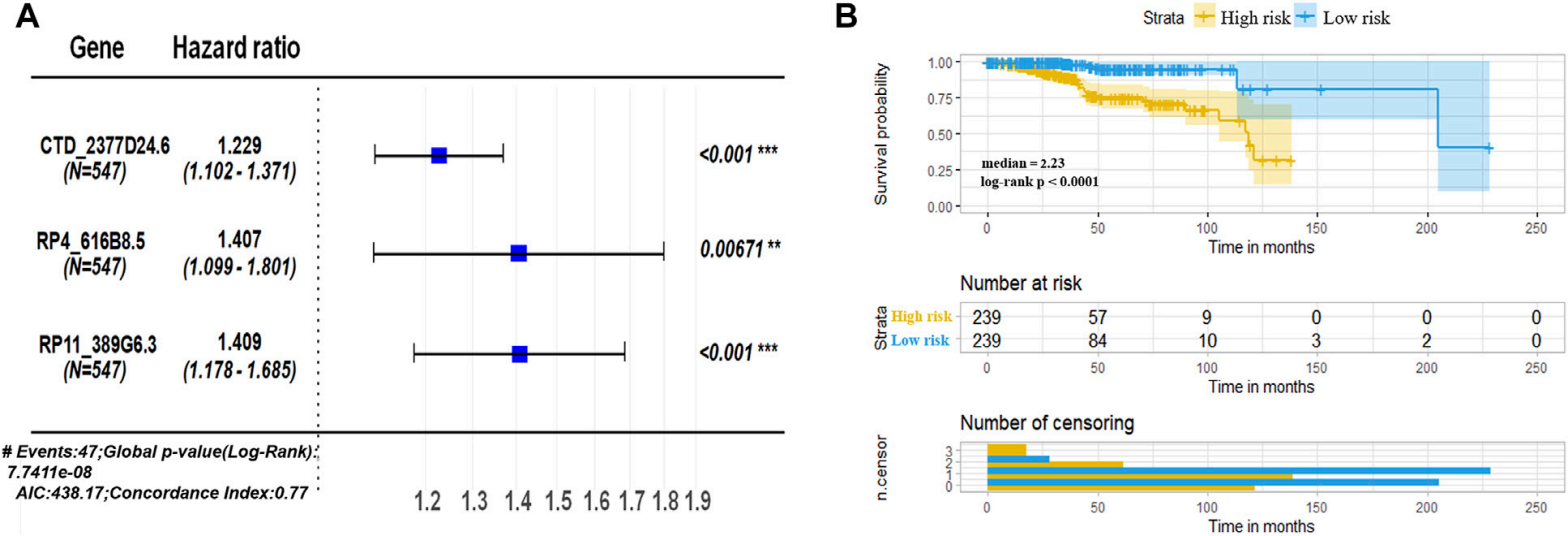
Assessment of prognostic risk in UCEC patients using a 3-lncRNA model. **(A)** Hazard ratios of the signature for overall survival of 3 lncRNAs. **(B)** Kaplan–Meier plot of OS according to risk score of the 3-lncRNA signature in TCGA–UCEC. TCGA patients were divided into high risk (3-lncRNAs expressions higher than median) and low risk (3-lncRNA expressions lower than median), classified according to formula of signature, CTD-2377D24.6 expression × 0.206 + RP4-616B8.5 × 0.341 + RP11-389G6.3 × 0.343.

To better identify the prognostic signature for UCEC, 3 lncRNAs (hazard ratios for CTD-2377D24.6 = 1.229, RP4-616B8.5 = 1.407, and RP11-389G6.3 = 1.409) with the lowest *p*-value (*p* < 0.1) were picked out for further investigation (Figure 3A). Based on the coefficients of 3 prognostic lncRNAs from multivariate Cox regression analysis (Liu Y et al., 2019; Jiang et al., 2021), the risk score of the 3-lncRNA signature for OS was identified as CTD-2377D24.6 expression × 0.206 + RP4-616B8.5 × 0.341 + RP11-389G6.3 × 0.343. According to the median cutoff value of this prognostic signature, patients were divided into low-risk and high-risk sets. The survival results demonstrated that the low-risk set had a higher survival rate than that of the high-risk set (*p* < 0.0001, Figure 3B). To assess the potential prediction of 3-lncRNAs for overall survival of UCEC patients, the AUC analysis was performed to test the 3-lncRNA signature compared with each lncRNA. The results showed that the 3-lncRNA signature insignificantly showed an excellent performance than that of each lncRNA and two lncRNAs (Supplementary Figure S1, S2).

### Correlation Between the 3-Long Noncoding RNA Signature and Clinical Characteristics of The Cancer Genome Atlas–Uterine Corpus Endometrial Carcinoma

To better understand the prognostic value of the 3-lncRNA signature, we further evaluated the relationships between the 3-lncRNA signature and traditional clinical characteristics. According to the median expressions of CTD-2377D24.6, RP4-616B8.5, RP11-389G6.3, and the 3-lncRNA signature risk score, the UCEC samples were divided into two sets. Pearson chi-square or Fisher’s exact tests revealed that the 3-lncRNA signature was closely linked with histological subtype (*p* < 0.0001), advanced clinical stage (*p* = 0.011), and clinical-grade (*p* < 0.0001) (Table 1). Compared to low-risk sets, the high-risk set tended to be serous adenocarcinoma (SAC), a histopathological type with worse differentiation and distant metastasis. These results demonstrated that 3-lncRNA signature was closely related to the conventional prognostic indicators.

**TABLE 1:** Relationships between 3-lncRNA set with histological subtype, clinical stage, and grade of EC patients.

Characteristics	RP11-389G6.3			RP4-616B8.5			CTD-2377D24.6			3-lncRNA signature		
	Low	High	*p value*	Low	High	*p value*	Low	High	*p value*	Low	High	*p value*
Histological subtype	—	—	—	—	—	—	—	—	—	—	—	—
EAC	235	174	0.0013	233	176	<0.0001	246	163	<0.0001	242	167	<0.0001
SAC	46	70	—	30	86	—	25	91	—	25	91	—
Other	9	14	—	11	12	—	9	14	—	7	16	—
Menopause status	—	—	—		—	—	—	—	—	—	—	—
Pre	40	29	0.4108	48	21	0.001	48	21	0.001	53	16	<0.0001
Post	237	213	—	217	233	—	217	233	—	213	237	—
Clinical stage	—	—	—	—	—	—	—	—	—	—	—	—
I	192	149	0.1841	198	143	<0.0001	187	154	0.0242	188	153	0.0111
II	26	25	—	15	36	—	26	25	—	21	30	—
III	60	67	—	50	77	—	59	68	—	56	71	—
IV	12	17	—	11	18	—	8	21	—	9	20	—
Grade	—	—	—	—	—	—	—	—	—	—	—	—
G1	62	36	0.0002	76	22	<0.0001	62	36	<0.0001	73	25	<0.0001
G2	77	43	—	83	37	—	78	42	—	79	41	—
G3 and high grade	151	179	—	115	215	—	140	190	—	122	208	—

EAC, endometrioid adenocarcinoma; SAC, serous adenocarcinoma.

### Expressions of 3 Long Noncoding RNAs in Paracancerous and Tumor Tissues of Uterine Corpus Endometrial Carcinoma Patients

**FIGURE 4:**
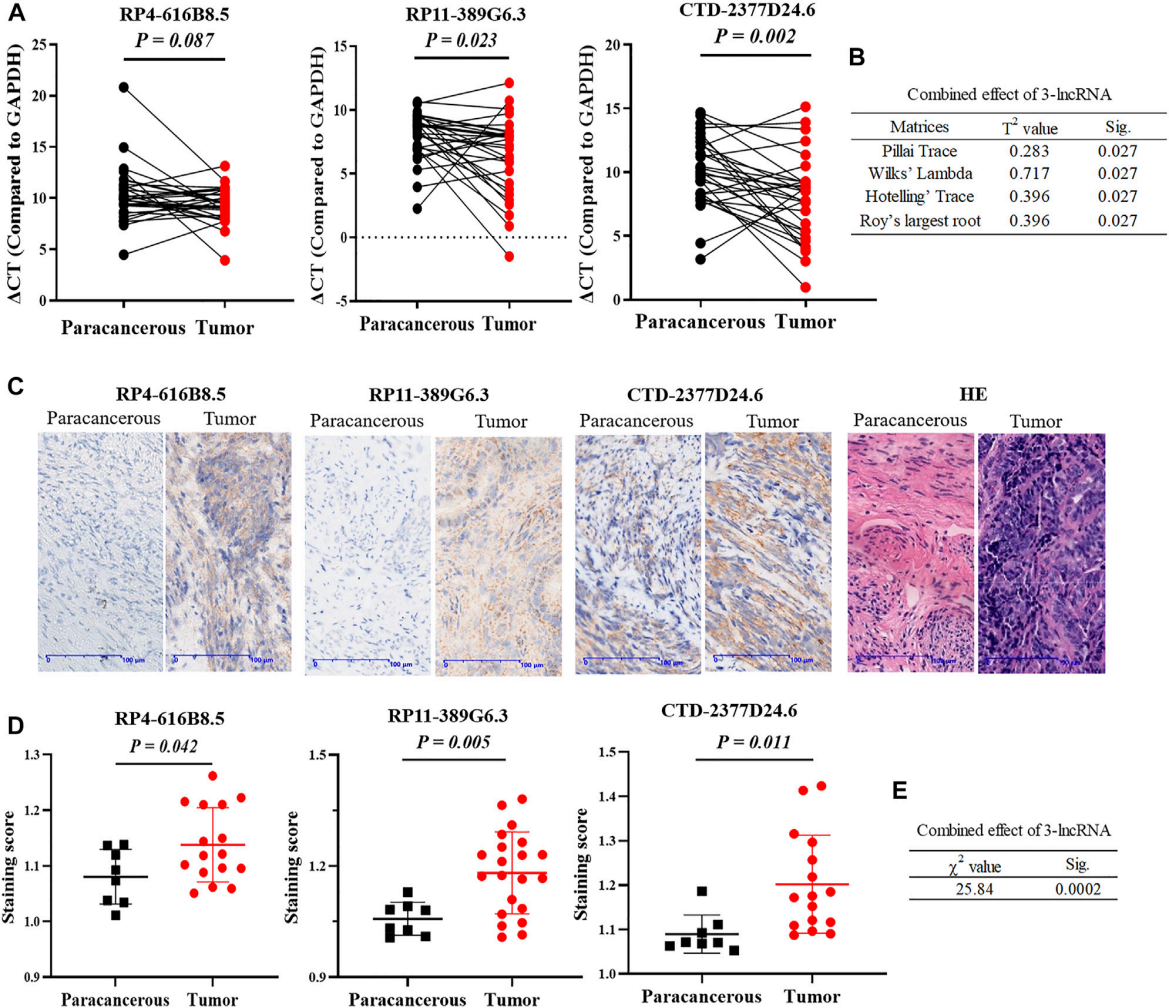
Validation of 3 lncRNAs expressions in clinical UCEC samples. **(A)** lncRNA RP4-616B8.5, RP11-389G6.3, and CTD-2377D24.6 mRNA expression in 30 paired paracanerous and tumor tissues of UCEC patients from the First Affiliated Hospital of Soochow University. **(B)** The combined effect of 3-lncRNAs using the Hotelling T2 test. **(C)** Representative images of *in situ* hybridization. **(D)** Staining scores of lncRNA RP4-616B8.5, RP11-389G6.3, and CTD-2377D24.6 levels in UCEC tissues and adjacent tissues by *in situ* hybridization. All data were presented as the mean ± SD. **(E)** The combined effect of 3-lncRNAs using Fisher’s method.

In addition, we validated the expressions of 3 lncRNAs in 30 paired paracancerous and tumor tissues of UCEC patients. First, the transcript abundances of RP4-616B8.5, RP11-389G6.3, and CTD-2377D24.6 were evaluated by qRT-PCR, and the results indicated that the expressions of RP11-389G6.3 and CTD-2377D24.6 were significantly higher in tumor tissues with *p*-values of 0.023 and 0.002, respectively, while the expression of RP4-616B8.5 did not show significant difference between tumor and paracancerous tissues with a *p*-value of 0.087 (Figure 4A), and the *p*-value of combined 3-lncRNAs was 0.027 using Hotelling T^2^ test (F = 3.56) (Figure 4B). Furthermore, *in situ* hybridization assay was also utilized to confirm the expressions of lncRNAs in paracancerous and tumor tissues of UCEC patients (Figure 4C). The staining scores of RP4-616B8.5, RP11-389G6.3, and CTD-2377D24.6 in EC tissues were significantly higher than those in paracancerous tissues with *p*-values of 0.042, 0.005, and 0.011, respectively (Figure 4D), and the *p*-value of combined 3-lncRNAs was 0.0002 using the Fisher’s methods (χ^2^ = 25.84) (Figure 4E). These results revealed that 3-lncRNA signature exhibited a better performance than the independent 3 lncRNAs for EC diagnosis.

### Functional Analysis of 3-Long Noncoding RNA Signature in Uterine Corpus Endometrial Carcinoma

**FIGURE 5:**
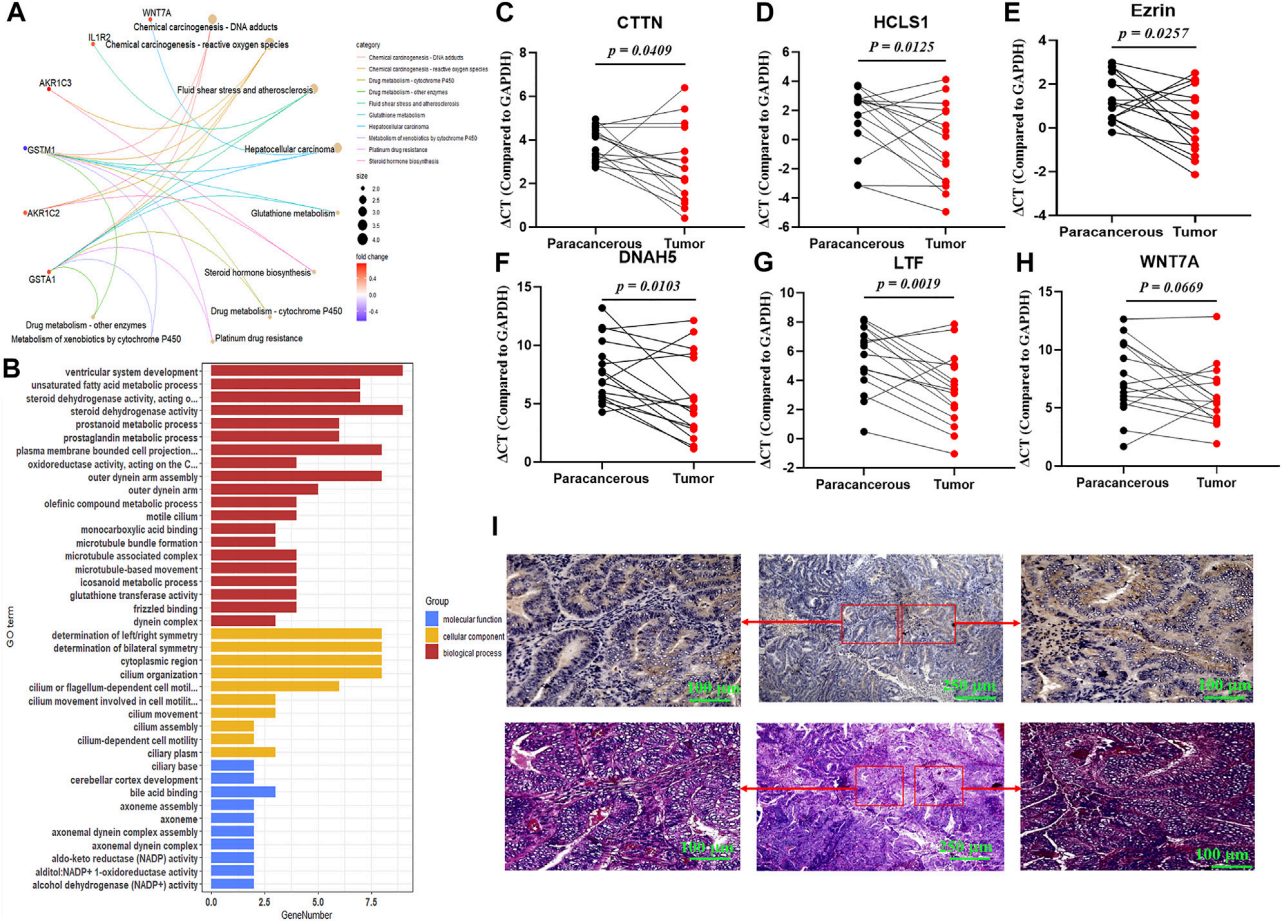
Functional analysis of 3-lncRNA signature. KEGG pathway **(A)** and Gene ontology **(B)** analysis (*p* < 0.05) of the 3-lncRNA signature related functional mRNAs. **(C-H)** qRT-PCR of indicated DeRNA expressions in 16 paired paracanerous and tumor tissues of UCEC patients from the First Affiliated Hospital of Soochow University. **(I)** Representative images of cortactin expression and location using immunohistochemical staining in UCEC tissues.

To explore the potential roles of 3-lncRNA signature in UCEC, differentially expressed mRNAs (DeRNAs) between the high-risk (3-lncRNAs at high levels) and low-risk (3-lncRNAs at low levels) groups were identified in endometrium carcinoma patients (Supplementary Table S2), and KEGG and GO analysis were conducted. Functional enrichment analysis revealed that these DeRNAs were significantly enriched in 5 KEGG pathways, including carcinogenesis, drug metabolism and resistance, fluid shear stress, and steroid hormone biosynthesis (Figure 5A), 20 GO terms in biological processes, 10 GO terms in cellular components, and 10 GO terms in molecular functions (Figure 5B), indicating that drug metabolism, chemical carcinogenesis, and cell motility–related pathways might be involved. Given the importance of cortactin for invadopodia formation, cancer cell migration, and metastasis (Ji et al., 2020), we examined the mRNA expression levels and location of cortactin by qRT-PCR and immunohistochemical staining. The results showed that two cortactin-encoding genes, CTTN and HCLS1, were markedly increased in tumor tissues (Figures 5C,D). Interestingly, immunohistochemical staining revealed that cortactin exhibited in gland duct cells, but not in supporting cells (Figure 5I). In addition, the greatest differentially expressed mRNAs between the high-risk (3-lncRNA signature at high levels) and low-risk (3-lncRNA signature at low levels) groups were also determined. DNAH5, LTF, and Ezrin were significantly increased in tumor tissues (*p* < 0.05, Figures 5E–G), and WNT7A displayed a slight increase (*p* = 0.0669, Figure 5H). These results indicated that cortactin might be associated with the function of 3-lncRNA signature.

## Discussion

Currently, a growing number of literatures demonstrated that dysregulated lncRNAs were involved in various diseases, as well as cancers (Evans et al., 2016). lncRNA might be a promising biomarker for cancer diagnosis, treatment, and prognosis prediction. Due to the heterogeneity of the tumor, a panel of lncRNA signature was more precise than a single lncRNA. In the present study, using Cox and LASSO regression, a 3-lncRNA signature was identified for predicting OS of EC patients. According to the median cutoff value of 3-lncRNA model, we demonstrated that the high-risk set displayed a poor survival, a higher clinical stage, and clinical grade and tended to be serous adenocarcinoma, a histopathological type with worse differentiation and distant metastasis. Our clinical samples also confirmed that 3-lncRNA, RP4-616B8.5, RP11-389G6.3, and CTD-2377D24.6 levels were increased in EC tissues than in paracancerous tissues by qRT-PCR and *in situ* hybridization. These findings provide an important hint that the 3-lncRNA signature has the potential performance for EC diagnosis and prognosis.

Mechanically, lncRNAs play a crucial role in EC progression by multiple patterns such as signaling, decoying, scaffolding, and guidance (Liu H et al., 2019). For example, Dong et al. reported that DLEU2 potentially interacted with miR-455 and miR-181a to promote epithelial-to-mesenchymal transition (EMT) and aerobic glycolysis (Dong et al., 2021). NEAT1 initiates a miR-361–mediated network to drive aggressive EC progression (Dong et al., 2019). lncRNA SOCS2-AS1 was found to suppress EC progression by promoting Aurora kinase A (AURKA) degradation via the ubiquitin–proteasome pathway (Jian et al., 2021). Additionally, Zhao et al. revealed that DLX6-AS1 promoted EC progression by recruiting p300/E2F1 in the DLX6 promoter region (Zhao and Xu, 2020). However, we found that little was known about the roles of RP4-616B8.5, RP11-389G6.3, and CTD-2377D24.6 in tumorigenesis and progression. One study reported that CTD-2377D24.6 was significantly induced by heat shock (Kim et al., 2020). Another study showed that CTD-2377D24.6 was a predictive factor in HCC patients with cirrhosis (Ye et al., 2020). No public study reported other two lncRNAs (RP4-616B8.5 and RP11-389G6.3). In the present study, we verified that the expressions of RP4-616B8.5, RP11-389G6.3, and CTD-2377D24.6 were higher in EC tissues than paracancerous tissues by qRT-PCR and *in situ* hybridization assays. According to the median cutoff value of 3-lncRNA signature, low-risk and high-risk sets were divided, and DeRNAs were identified. KEGG and GO analysis found that drug metabolism, chemical carcinogenesis, and cell motility–related pathway were enriched, indicating the potential roles of a panel of lncRNAs in initiation, metastasis, and chemoresistance of EC. The extensive quantity of published reports suggested that cell motility at an early stage in cancer correlated with metastasis (Lambert et al., 2017). In particular, the importance of cortactin for invadopodia formation, cancer cell migration, and metastasis has been proven (Schnoor et al., 2018; Ji et al., 2020). However, the link between lncRNAs and cortactin in endometrial carcinoma remains unclear. Here, we demonstrated that cortactin was markedly increased in UCEC tumor tissues, and especially exhibited in gland duct cells. In addition, the greatest differentially expressed mRNAs between the high-risk and low-risk groups, such as DNAH5, LTF, and Ezrin, were significantly increased in tumors. These hinted that more comprehensive studies about the molecular mechanism of 3-lncRNAs will remain to be lucubrated.

Traditional therapeutic strategies and risk stratification for EC patients are based on clinical and histological characteristics. However, the conventional classification does not adequately depict tumor biology owing to the high heterogeneity of EC. Recently, molecular or genomic classification has drawn attention as a promising approach to predict cancer prognosis. Levine et al. assessed the genome, transcriptome, and proteome of 373 endometrial carcinomas. Based on integrated genomic data, they were classified into four subgroups: POLE ultramutated, microsatellite instability hypermutated, copy-number low, and copy-number high. Subsequently, another molecular classification for EC termed “ProMisE” was identified (Talhouk et al., 2015). A similar integrated risk profile was established by the TransPORTEC international consortium (Stelloo et al., 2015; Stelloo et al., 2016). However, compared with genome sequencing, our 3-lncRNA signature was more suitable for clinical diagnosis and classification due to its higher stability and lower cost.

By bioinformatic approaches and verification of clinical samples, we demonstrated that the 3-lncRNA signature might be a reliable prognostic biomarker. However, there are several limitations in our study. First, the 3-lncRNA signature was constructed by the TCGA–UCEC datasets, in which the Caucasian race was the main patient. So, the prognostic value in other races is needed to be validated. Second, we detected the independent difference of 3 lncRNAs between the paracancerous tissues and UCEC tissues in our clinical samples, while the prognostic value of the signature was not analyzed due to insufficient prognostic data. Third, the 3 lncRNAs were rarely reported, and their potential function was unclear. Although functional enrichment analysis based on the DeRNAs in high- and low-risk signatures was performed, the potential mechanisms should be further experimentally investigated.

In conclusion, we revealed a potential 3-lncRNA signature that could accurately predict outcomes for UCEC patients. Meanwhile, we found that the 3-lncRNA signature was closely associated with clinical characteristics. Furthermore, we validated the different expressions of the 3 lncRNAs in our clinical samples, indicating that a panel of 3-lncRNAs exhibited better performance for EC diagnosis. These findings provide the first hint that the set of lncRNAs may play a critical role in the initiation and metastasis of EC, indicating a new signature for early diagnosis and therapeutic strategy of uterine corpus endometrial carcinoma.

## Materials and Methods

### Data Source and Differentially Expressed Long Noncoding RNA Screening

The lncRNA expression data and corresponding clinical information of UCEC patients were open-accessed from TCGA data portal (https://www.cancer.gov/tcga), including 548 UCEC tissues and 35 normal tissues. The differentially expressed lncRNAs (DElncRs) were identified by using the R package “DEseq,” “edgeR,” and “limma” with |Log2 fold-change (FC)| > mean|Log2FC| ± 2 × sd |Log2FC| and adjusted *p* < 0.05. A volcano plot of DElncRs was obtained by using the R package (Anders and Huber, 2010; Robinson et al., 2010; Ritchie et al., 2015).

### Construction and Assessment of Long Noncoding RNA-Based Prognostic Signature

First, univariate COX regression and least absolute shrinkage and selection operator (LASSO) model were selected to find the independent prognostic lncRNAs. The LASSO method was performed by the package “glmnet” in the R software. Subsequently, multivariate Cox regression analysis was used to construct the prognostic signature. A risk score formula was constructed as follows: gene 1 × b1 + gene 2 × b2 + gene 3 × b3 +···gene n × bn, in which b represented the respective coefficient of genes. Gene represented the expression level of each gene. Subsequently, the risk score of prognostic signature formula was calculated as follows: CTD-2377D24.6 expression × 0.206 + RP4-616B8.5 × 0.341 + RP11-389G6.3 × 0.343. According to the median of risk score, the TCGA–UCEC patients were divided into the high-risk set and the low-risk set. To evaluate the prognostic signature of lncRNAs, the Kaplan–Meier and time-dependent receiver operating characteristic (ROC) curve analysis were performed.

### Functional Enrichment Analysis

Kyoto Encyclopedia of Genes and Genomes (KEGG) and Gene Ontology analysis were performed with the “clusterProfiler” R package to identify the function of lncRNA-based signature (Yu et al., 2012). Significant functional categories were filtered into the meaning of *p*-value and false discovery rate (FDR) values <0.05.

### RNA Extraction and Quantitative Real-Time Polymerase Chain Reaction

Total RNA was extracted using TRIzol (Life Technologies, NY, United States). The concentration and integrity of RNA were verified by a NanoDrop spectrophotometer (Thermo Fisher Scientific, Waltham, MA, United States). Afterward, the total RNA was reverse-transcribed into cDNA using the PrimeScript RT reagent kit (Takara, Dalian, China). The expressions of 3 lncRNAs were measured by qRT-PCR using the Hiff qPCR SYBR Green Master Mix (Yeasen Biotech Co., Shanghai, China) in the QuantStudio 6 system (Applied Biosystems, Waltham, MA). The primers synthesized are listed in Table 2.

**TABLE 2:** Primer sequences for qRT-PCR.

Primers	Sequence (5’to 3′)
CTD-2377D24.6-F	TTC​CGG​TGT​CCA​GAT​GTT​CA
CTD-2377D24.6-R	AAG​GTG​AGT​TGG​GGA​GGA​TG
RP4-616B8.5-F	ATG​AGT​GTG​GCA​GCC​TAT​GT
RP4-616B8.5-R	AAC​TCC​TGA​CCT​CGT​GAT​CC
RP11-389G6.3-F	GGC​CTT​GAG​AGA​TAG​AGG​GG
RP11-389G6.3-R	ATA​CGT​CCT​TCC​CAT​CCT​GC
DNAH5-F	GAG​GCA​GAG​TCA​CTG​ACG​AC
DNAH5-R	TCT​CAT​CCC​CTC​CAC​CAG​AG
WNT7A-F	CTG​GGC​ATG​GTC​TAC​CTC​CG
WNT7A-R	GGC​CAT​TGC​GGA​ACT​GAA​AC
LTF-F	GTC​CCT​TCT​CAT​GCC​GTT​GT
LTF-R	CCT​TTC​AGC​ACC​AGG​GCG​A
Ezrin-F	GGA​TAA​GAA​GGT​GTC​TGC​CCA
Ezrin-R	TCC​CAC​TGG​TCC​CTG​GTA​AG
CTTN-F	ATG​TCA​CCC​AGG​TGT​CCT​CT
CTTN-R	AAG​CCG​CAT​CCT​CAT​AGA​CG
HCLS1-F	TGA​GTA​TGT​TGC​CGA​GGT​GG
HCLS1-F	CTC​GTG​TTT​CTC​CGT​CTC​TCC
GAPDH-F	GCA​CAG​TCA​AGG​CTG​AGA​ATG
GAPDH-R	ATG​GTG​GTG​AAG​ACG​CCA​GTA

### Ethics Statement

Publicly available TCGA datasets were analyzed in this study, and approval from a local Ethics Committee was not necessary. For human subjects, all procedures were carried out according to Helsinki Declaration and institutional guidelines and were approved by the Ethics Committee at the First Affiliated Hospital of Soochow University.

### *In situ* Hybridization Assay

The paraffin embedded UCEC and adjacent normal tissues were stained to detect the lncRNA expression. The lncRNA probes were designed and produced by SimaifuBio (Suzhou, Jiangsu, China). The probe sequences are presented in Table 3. In brief, sections were deparaffinized, digested, and blocked with 3% methanol-H_2_O_2_; then, the sections were dropped with prehybridization solution and incubated for 1 h in the incubator at 37°C. With the absorption of the excess liquid, the hybridization solution containing indicated lncRNA probes was added and then incubated in the incubator at 42°C overnight. Next day, after washing, the samples were dropped with block solution and incubated for 30 min at room temperature. After that, digoxigenin-labeled peroxidase antibody was added to incubate for 40 min in the incubator at 37°C. Afterward, the sections were added with DAB coloration, and the positive signal appeared brown–yellow. Hematoxylin staining solution was used to stain the nucleus. CaseViewer 2.2.1 (3DHISTECH Ltd.) and Image Pro Plus 6 were used for image capture and analysis, respectively.

**TABLE 3:** Probes sequences for *in situ* hybridization.

Probe	Sequence (5’to 3′)
CTD-2377D24.6	CCC​UUA​CCC​ACG​GGU​GAC​AGC​CAU​UUU​GAG
RP11-389G6.3	GAG​ACA​GGA​GUU​UGG​GGC​UGA​UGG​GCU​UGG
RP4-616B8.5	GAA​GAG​CAG​GCA​GUU​UUU​CUG​GUU​UUU​GAG​GUU​AG

### Statistical Analysis

All of the expression profiles and clinical information were obtained from TCGA by R software. All statistical analyses were carried out using SPSS23.0 (SPSS, Chicago, IL, United States) or R software. For continuous variables, Student’s t-test was used to compare the difference between the two groups. For categorical variables, χ^2^ test was used to compare the differences among groups. Fisher’s method and Hotelling T2 test were used to combine *p* value. *p* < 0.05 was considered to be statistically significant.

## Data Availability

The original contributions presented in the study are included in the article/Supplementary Material, further inquiries can be directed to the corresponding authors.
