# Supplementary material for: Prediction of Clinical Outcome in Endometrial Carcinoma Based on a 3-lncRNA Signature
Source: Front Cell Dev Biol. 2022 Feb 1;9:814456. doi: 10.3389/fcell.2021.814456 (PMC8844015; doi:10.3389/fcell.2021.814456)
Supplement: Supplementary file 1 [file DataSheet1.PDF]

Supplementary Data

The prediction of clinical outcome in endometrial carcinoma based on a 3-lncRNAs signature

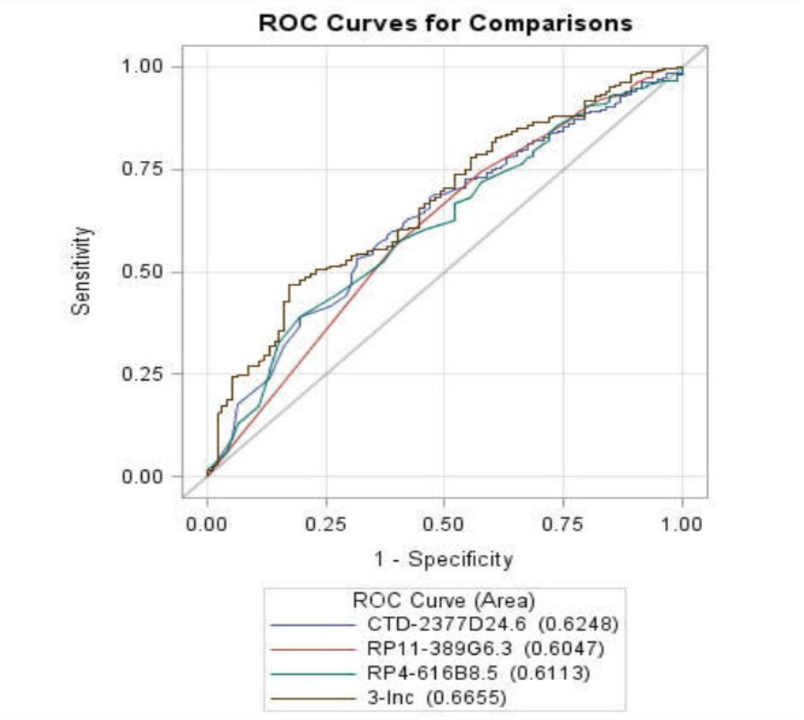

Figure S1. The comparison between 3-lncRNAs and each lncRNA by ROC analysis. The AUC for 3-lncRNAs was 0.67 (95%CI = 0.61-0.72), RP4-616B8.5 was 0.61 (95%CI = 0.55-0.67, *p* = 0.06), RP11-389G6.3 was 0.60 (95%CI = 0.54-0.67, *p* = 0.01), and CTD-2377D24.6 was 0.62 (95%CI = 0.56-0.69, *p* = 0.11), respectively.

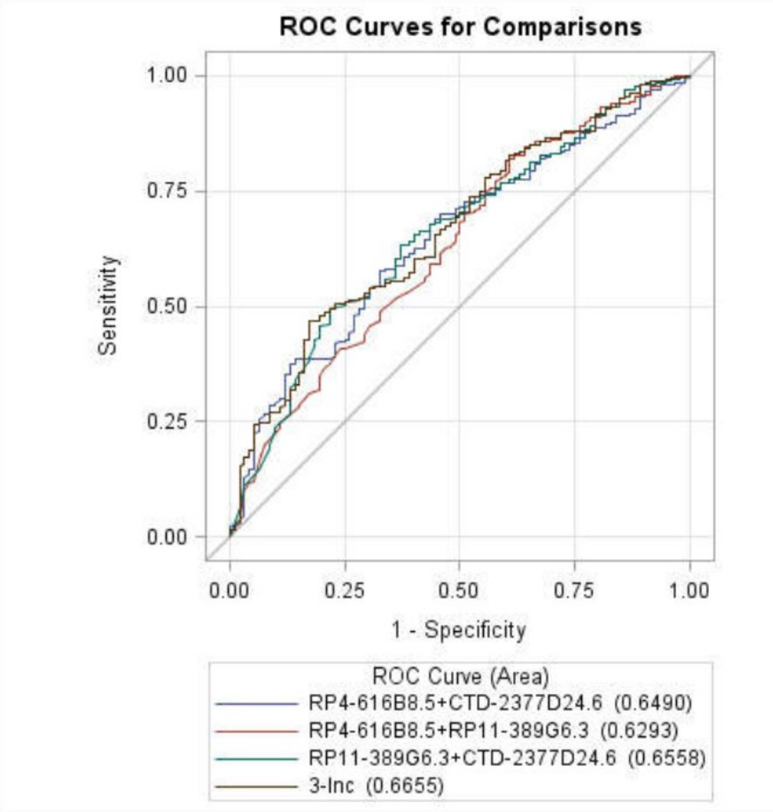

Figure S2. The comparison between 3-lncRNAs and each 2-lncRNA by ROC analysis. The AUC for 3-lncRNAs was 0.67 (95%CI = 0.61-0.72), the AUC for combination of RP4-616B8.5 and CTD-2377D24.6 was 0.65 (95%CI = 0.59-0. 71, *p* = 0.36), for combination of RP11-389G6.3 and CTD-2377D24.6 was 0.66 (95%CI = 0.57-0.69, *p* = 0.09), and the combination for RP4-616B8.5 and RP11-389G6.3 was 0.63 (95%CI = 0.56-0.69, *p* = 0.42), respectively.
